# Single-section multiplex spatial proteomics of immune microenvironments in kidney transplantation

**DOI:** 10.3389/fimmu.2026.1861589

**Published:** 2026-08-24

**Authors:** Deborah Mattinzoli, Carlo Alfieri, Masami Ikehata, Silvia Armelloni, Paolo Molinari, Simona Verdesca, Anna Regalia, Silvia Malvica, Francesca Zanoni, Margherita Di Naro, Min Li, Nicholas Walter Delfrate, Antonella Tosoni, Manuela Nebuloni, Alessandro Del Gobbo, Manuel Alfredo Podestà, Giuseppe Castellano

**Affiliations:** 1 Renal Research Laboratory, Fondazione IRCCS Ca’ Granda Ospedale Maggiore Policlinico, Milan, Italy; 2 Unit of Nephrology, Dialysis and Renal Transplantation, Fondazione IRCCS Ca’ Granda Ospedale Maggiore Policlinico, Milan, Italy; 3 Department of Clinical Sciences and Community Health, University of Milan, Milan, Italy; 4 Post-Graduate School of Specialization in Nephrology, University of Milan, Milan, Italy; 5 Pathology Unit, ASST Fatebenefratelli-Sacco, Milan, Italy; 6 Division of Pathology, Fondazione IRCCS Ca’ Granda Ospedale Maggiore Policlinico, Milan, Italy

**Keywords:** deep phenotyping, fibrosis, kidney disease, multiplexed spatial proteomics, spatial immune profiling, transplantation

## Abstract

Characterizing kidney disease is challenged by marked cellular heterogeneity and limited tissue availability from renal biopsies. Conventional diagnostic workflows rely on multiple serial sections for parallel staining, increasing tissue consumption, sampling bias, and loss of spatial information, thereby constraining molecular characterization within intact tissue architecture. High-plex spatial proteomics may overcome these limitations by enabling comprehensive molecular profiling on a single section. Here, we present and evaluate a high-plex cyclic immunofluorescence imaging workflow (MACSima™, Miltenyi Biotec) applied to kidney transplant biopsies, including BK virus nephropathy (BKVN) and focal segmental glomerulosclerosis (FSGS), to characterize spatial immune organization with a focus on complement system components. Feasibility and subcellular resolution were first assessed in a lupus nephritis section, demonstrating compatibility with diagnostic immune panels and preservation of tissue morphology. A 48-marker multiplex panel interrogating immunity, oxidative stress, senescence, and fibrosis was then applied to BKVN samples, including paired pre- and post-treatment biopsies, revealing distinct proteomic patterns and dynamic changes following therapy. In FSGS, a glomerulus-focused panel identified spatially resolved innate and adaptive immune signatures, including complement-related patterns supporting exploratory analysis of glomerular immune architecture. Structural, nuclear, membrane, and phosphorylated signaling markers enabled precise delineation of renal compartments and assessment of cellular states such as proliferation, DNA damage, and pathway activation. The workflow also supported detection of extracellular vesicles in cultured renal cells, highlighting its versatility. Overall, this approach provides a robust, tissue-sparing platform for integrated spatial and molecular profiling of renal biopsies, reducing sampling bias while enabling discovery-level phenotyping from a single section. This unified strategy is particularly suited to kidney transplantation, where diagnosis, therapeutic decision-making, and longitudinal monitoring are closely interconnected.

## 1 Introduction

Despite substantial advances driven by omics-based genetic discoveries, translational progress in nephrology particularly in mechanistic understanding and clinical application has historically lagged behind the progress achieved in oncology and hematology (1). A major limitation is sample availability: the invasive nature of kidney biopsy (KBx) restricts its use in critically ill patients and in post-transplant (KTx) monitoring, while the need for multiple assays increases tissue consumption and variability. In addition, formalin-fixed paraffin-embedded (FFPE) samples are often suboptimal for modern omics approaches, underscoring the need for improved processing, standardization, and collaborative frameworks (2).

The intrinsic structural complexity of the kidney, characterized by intricate vasculature, diverse cell populations, and tightly regulated neuro-hormonal networks, further complicates molecular analysis (3). As a result, bulk omics approaches lack sufficient spatial resolution, prompting the use of laser capture microdissection (LCM) to isolate specific compartments. However, LCM remains limited by potential cross-contamination, low yield, RNA degradation, and loss of spatial context (4). Although recent FFPE-compatible RNA-seq methods have partially mitigated these issues, formalin-induced crosslinking continues to constrain transcript length and coverage (5). Consequently, despite their impact in other fields, transcriptomic and miRNA-based approaches remain underutilized in nephrology (6, 7). Less invasive strategies, such as liquid biopsy, including extracellular vesicles (EVs), cell-free DNA/RNA, and circulating miRNAs, offer promising alternatives for monitoring kidney injury and allograft rejection; however, they currently lack the spatial context provided by tissue-based approaches (8–10). Nevertheless, renal biopsy remains the gold standard for definitive diagnosis (11).

While nephrology has advanced considerably in genetic diagnostics, with gene discoveries reshaping disease classification (12), mechanistic insights have lagged due to a persistent gap between genotype and phenotype (13). In this context, proteomics provides a more direct readout of biological processes compared to transcriptomics, capturing functional molecular states rather than potential gene expression outcomes (14).

Emerging spatial proteomic approaches, including LCM-DIA, microPOTS, and top-down mass spectrometry, have begun to address this gap by enabling protein profiling in defined tissue regions. However, these methods still rely on tissue dissociation or microdissection, limiting preservation of native architecture and often resulting in reduced spatial resolution, sensitivity constraints, and artefacts related to formalin-induced crosslinking (15–17).

Together, these limitations highlight the need for spatially resolved proteomic strategies capable of preserving tissue architecture while enabling *in situ* investigation of disease-specific mechanisms within renal microenvironments. Here, we present MACSima Imaging Cyclic Staining (MICS), a high-plex spatial proteomics platform designed to address current diagnostic and research limitations in kidney biopsy analysis and to support applications in transplantation and chronic kidney disease.

We adopted a stepwise approach, progressing from diagnostic validation to compartment-specific spatial analysis of the renal microenvironment. Given the exploratory nature of this proof-of-concept study, a limited number of samples was analyzed, and our primary objective was to establish and validate the methodological workflow demonstrating the feasibility of high-plex spatial proteomic analysis in renal tissue, rather than to draw definitive mechanistic or disease-specific biological conclusions.

We first investigated whether MICS could simultaneously reproduce routine diagnostic immunoprofiles on a single 3-µm KBx section, addressing the critical challenge of tissue scarcity while preserving tissue morphology and diagnostic information. This validation was performed using native kidney biopsies to demonstrate the feasibility of reproducing the routine diagnostic immunopanel on a single tissue section, irrespective of whether the biopsy originated from a native or transplanted kidney. A lupus nephritis (LN) biopsy with a classical full-house immune profile was included as a technical benchmark because it was positive for the complete panel of routine diagnostic markers. We then established a spatial reference map of the major renal structural compartments and functional markers in healthy and diseased kidney tissue.

We then assessed its ability to identify disease-associated patterns in KTx biopsies by analyzing protocol “normal” controls (ctrl-KTx) and BK virus nephropathy (BKVN), a condition primarily affecting the tubulointerstitial compartment, using a 48-marker panel. This approach was extended to a longitudinal BKVN case, enabling comparison of pre- and post-treatment biopsies (18). Spatial organization was further refined using a distance-based approach focused on fibrotic regions, enabling quantification of spatial immune gradients in tubulo-interstitial fibrosis.

To further refine compartment-specific analysis, we applied a 30-marker panel to recurrent focal segmental glomerulosclerosis (rFSGS), focusing on the glomerular compartment, where the spatial organization of innate and adaptive immunity, as well as complement system activity, remains incompletely understood. Spatial correlation analysis enabled exploratory reconstruction of immune interaction networks within the glomerular microenvironment. Finally, we demonstrate the platform’s versatility through subcellular imaging of extracellular vesicles (EVs) derived from plasma of KTx patients and applied to a podocyte model, highlighting its potential for integrated tissue and circulating biomarker characterization.

## 2 Materials and methods

### 2.1 Renal tissue collection and case selection

Kidney biopsies were collected from patients followed at the Adult Nephrology, Dialysis, and Transplant Units of the Fondazione Ca’ Granda IRCCS Ospedale Policlinico of Milan. A comprehensive summary of the patient cohorts, biopsy types, and clinical rationales for each experimental setting is provided **Supplementary Table 1**. To evaluate MICS performance for diagnostic immunoprofile reproduction and tissue preservation, we tried to compare two different patterns: the first from a normal control case, derived from nephrectomy specimens obtained during tumor excision, and the second from a LN biopsy, a condition of high positivity included as a technical validation because of its characteristic “full house” immunological pattern. The nephrectomy-derived sample served as a healthy control and was also used to demonstrate high-resolution structural mapping of the kidney. For the tubulo-interstitial compartment, three additional patient groups were studied: 1) Protocol “normal” allograft KBx (ctrl-KTx) without histologic abnormalities (n = 3); 2) Clinically indicated KBx with BKVN, defined by renal function decline, BK viremia, and urinary decoy cells (n = 3); 3) A paired BKVN pre-/post-therapy (Immunoglobulines 0,4 g/Kg repeated 6 times; stop MMF and EVR initiation). The patient examined is a 40-yrs-old patient. From late 2021 (1 yr of RTx), she developed BK polyomavirus viremia (>30000 copies/ml), initially managed by MMF withdrawal, switch to everolimus, and stepwise CNI minimization (tacrolimus → ciclosporin) plus two cycles of IVIG. Biopsy in January 2024 (sCr 1.1 mg/dL, BKVDNA 14000000 copies/ml, before IVIG therapy), showed BKVN class 3 with CNI tubular toxicity; a repeated post-treatment KBx, performed in April 2025 for a renal functional decline (sCr 1.5 mg/dL, BKVDNA 50000 copies/ml), showed chronic tubulo-interstitial damage with SV40 negativity, compatible with sequelae of BKVN. Despite partial virological response, BK viremia persists at low–moderate levels and creatinine is around 1.6–1.7 mg/dL. She is currently maintained on dual immunosuppression with prednisone and ciclosporin.

In addition, 2 KTx from patients with rFSGS were included to perform glomerulus-focused spatial analysis of innate and adaptive immune pathways, including complement system markers, for compartment-specific characterization of immune and functional markers.

The study was approved by the local ethics committee (protocol numbers OSMAMI-17/12/2021-0050939-U and 4759-1837/19) and was conducted in accordance with the Declaration of Helsinki.

### 2.2 Standard diagnostic histology and immunohistochemistry

The KBx were FFPE following the hospital’s standard protocol. Samples were fixed in 4% paraformaldehyde for 30 minutes, rinsed in distilled water (dH_2_O) for 5 minutes, and dehydrated through a graded ethanol (EtOH) series: 70%, 80%, 95% EtOH, and 100% EtOH, followed by clearing in xylene (every 10 minutes × 3 times). Sections of 3 μm thickness were dewaxed using the following protocol: xylene (2 minutes x 2 times) followed by EtOH 100% (2 minutes x 2 times), 80%, 70%, 50% and rinsed in dH_2_O (2 minutes each). For Periodic Acid–Schiff (PAS) staining, sections were incubated with Schiff’s reagent for 20 minutes at 45 °C and counterstained with hematoxylin for 12 minutes (Roche, Monza, Italy). For Acid Fuchsine Orange G (AFOG) staining, procedures were carried out according to the manufacturer’s instructions (DIAPATH, Bergamo, Italy). For Hematoxylin–Eosin (H&E) staining (both reagents from BIOPTICA, Milan, Italy), the sections were stained with hematoxylin for 9 minutes, rinsed under running water for 6 minutes, and counterstained with eosin for 1 minute. For IgA, IgG, IgM, C1q, C3c, fibrinogen, and κ/λ chain, the antibodies were incubated for 1h after the antigen retrieval carried out with enzymatic pronase digestion (37 °C, 50 minutes). The full list of markers, including their biological functions and rationale for inclusion in the panel, is provided in **Supplementary Table 2**.

### 2.3 MICS technology

Spatial proteomics was performed using the MACSima™ Imaging System (Miltenyi Biotec, Bergisch Gladbach, Germany), a fully automated platform combining liquid handling and widefield microscopy for cyclic immunofluorescence imaging. The FFPE kidney biopsy (KBx) was processed according to the hospital’s standard protocol. Sections (3 µm thick) were mounted on SuperFrost Plus slides supplemented with an additional adhesive coating (Vector Laboratories, CA, USA) and incubated overnight at 40 °C. Before staining, sections were dewaxed by the following protocol: xylene (20 minutes), 100% EtOH (1 minute), 95% EtOH (1 minute), ending with dH_2_O (1 minute). Then, Antigen retrieval was carried out in TEC buffer (Dako, Agilent Technologies, Milan, Italy), pH 9, at 98 °C for 20 min (instead of standard enzymatic pronase) followed by permeabilization with 0.2% Triton X-100 for 10 min. Slides were then mounted in a MACSwell™ Imaging Frame (130-124-675, Miltenyi Biotec) and pre-stained with DAPI (1:5 dilution, 130-127-574, Miltenyi Biotec) in MACSima Running Buffer (130-121-565, Miltenyi Biotec) for 10 min, washed three times, and adjusted to a final volume of 475 µL. Fluorochrome-conjugated primary antibodies were prepared in a MACSwell™ Deepwell Plate (130-126-865, Miltenyi Biotec) using Running Buffer and sealed with MACSwell™ Sealing Foil (130-126-866, Miltenyi Biotec) to prevent evaporation. DAPI (1:50 dilution) was incorporated into the antibody mix every eighth staining cycle. The full list of markers, including their biological functions and rationale for inclusion in the panel, is provided in **Supplementary Table 2**

#### 2.3.1 Image acquisition and preprocessing

All fields of view (FoV) were acquired using the automated MACSima imaging pipeline, and raw image stacks were processed with MACS iQ View software (v1.3.2, Miltenyi Biotec). Images were pre-aligned and quality-checked to ensure consistency and reproducibility across all fields of view.

#### 2.3.2 Cutoff strategy

To classify cells as positive or negative for each marker, mean fluorescence intensity (MFI) values were extracted from all segmented cells and visualized as intensity histograms. During image acquisition and post-processing, the platform automatically corrected for tissue autofluorescence and residual photobleaching. Since fluorescence intensity values showed predominantly unimodal distributions without clearly separated negative and positive cell populations, thresholds were defined using a histogram-based, background-driven approach rather than by separating distinct cell populations. Specifically, cutoffs were set slightly above the baseline fluorescence level to distinguish true marker-specific signals from background fluorescence. Thresholds were manually adjusted using the Miltenyi IQ View software, which allows simultaneous visualization of the fluorescence intensity histogram and the corresponding tissue image. This enabled verification that cells classified as positive displayed spatially consistent fluorescence patterns and the expected subcellular localization, while weak or diffuse signals attributable to background were excluded. Threshold selection was further confirmed in individual fields of view (FoVs) to ensure consistent identification of positive cells across tissue regions. The same thresholding criteria were consistently applied across samples for each marker to ensure comparability of fluorescence-based measurements. Colocalization with established markers and healthy KBx samples was used as an internal reference to confirm staining specificity and reduce potential false-positive assignments. This validation approach is particularly relevant for cyclic immunofluorescence analyses, where accurate interpretation of spatial protein expression requires both quantitative fluorescence measurements and visual assessment of tissue staining patterns; details on image processing are provided elsewhere (19). Because this background-driven approach mainly affects cells with fluorescence intensities close to the background level, whereas cells with clear marker expression remain consistently classified, small variations in cutoff placement are unlikely to substantially influence the overall interpretation of the data. Therefore, the results are based on reproducible differences in marker expression levels and spatial distribution across samples rather than on individual cells with weak or uncertain staining.

#### 2.3.3 Nucleus and cytoplasm segmentation

Cell segmentation enabled compartment-specific quantification of nuclear, cytoplasmic, and membrane-associated protein expression. Nuclei were segmented from DAPI images using the Advanced Morphology for Tissue algorithm, an automated image-analysis algorithm optimized for tissue morphology, with parameters optimized for renal tissue: minimum/maximum diameter of 30–70 µm, detection sensitivity of 200%, separation force of 75%, and smoothing sigma of 3.0. Cytoplasmic and membrane-associated markers were segmented using the Constrained Donut algorithm. Cytoplasmic markers included α-SMA, Cytokeratin 8/18, Desmin, HLA-DR, and Vimentin, while membrane-associated markers included β-Catenin, and CD20. Wheat Germ Agglutinin staining was incorporated to refine membrane boundaries and verify segmentation accuracy. Detection sensitivity and donut width were empirically optimized for each marker to minimize bleed-through and segmentation artifacts. Segmentation outputs were systematically cross-checked against raw images, known subcellular localization patterns, and healthy control tissue to ensure accuracy.

#### 2.3.4 ROI selection and spatial mapping

For tubulointerstitial analysis in BKVN cases, ten ROIs per case were selected from three protocol ctrl-KTx biopsies, three clinically indicated BKVN biopsies, and one paired pre- and post-treatment BKVN biopsy. Glomerular analysis was performed by tracing one ROI per glomerulus (12 in rFSGS, 11 in LN) using biopsies from two KTx-rFSGS, and two LN-KBx cases. Nuclear and cytoplasmic segmentation parameters were adjusted according to the tissue compartment for each ROI. Fibrosis niches were mapped using distance-based analysis of Collagen I deposition, the primary fibrillar collagen in renal interstitial fibrosis. Using the spatial analysis tools available in the MACSima imaging platform, regions enriched in Collagen I signal were identified as reference niches, and the spatial distribution of other markers was evaluated according to their relative distance from these positive areas. This distance-based approach enabled characterization of protein expression patterns within and around fibrotic regions, providing information on the spatial relationship between molecular markers and tissue remodeling niches.

#### 2.3.5 Quality control and validation

Quality control measures were implemented using positive control consisting of a full-house LN KBx to verify staining fidelity across all markers, and a healthy tissue region from a nephrectomy specimen to ensure specificity. Marker colocalization was used as an internal reference to confirm accurate subcellular localization and reduce false positives. Because the MICS platform automatically subtracts tissue-intrinsic autofluorescence and corrects residual photobleaching, additional unstained or isotype-matched negative controls were not required. All thresholds and segmentation outcomes were independently reviewed by at least two observers to ensure consistency and reproducibility.

#### 2.3.6 Quantitative Data Analysis and Visualization

Quantitative analysis of protein expression was performed using the MACS iQ View software (v1.3.2, Miltenyi Biotec), which enabled the extraction of MFI values, the calculation of the percentage of positive cells, and the assessment of expression changes for each marker across the selected ROIs. Fold-changes were calculated relative to control samples and expressed as log2 fold-change (log2FC) values for comparative analysis and visualization. Correlation matrices were generated from the quantitative expression values extracted from MACS iQ View, and correlation values greater than 0.7 were considered indicative of strong associations. Visualization of the data, including heatmaps of log2FC values, correlation matrices between markers, and distance-based mapping of fibrosis niches using Collagen I deposition was performed using the SRplot platform. All analyses were performed per ROI and replicated across multiple fields of view to ensure robustness and reproducibility.

### 2.4 Extracellular vesicles isolation, characterization, and functional analysis

Extracellular vesicles (EVs) are membrane-bound nanoparticles released by cells into biological fluids and recognized as important mediators of intercellular communication and potential biomarker sources. Their molecular composition, including proteins, lipids, and nucleic acids, reflects the characteristics of their cells of origin. In chronic kidney disease and antibody-mediated rejection, circulating EVs may provide insights into disease-associated cellular activation, injury, and interactions with recipient renal cells. EVs were isolated from the plasma of five patients with antibody-mediated rejection by differential ultracentrifugation using a Beckman Coulter L-100XP Optima ultracentrifuge (Beckman Coulter, CA, USA) and characterized according to MISEV guidelines to confirm their characteristics and sample quality. EV concentration, size, and morphology were assessed by Nanoparticle Tracking Analysis (NTA) using the Nanosight NS300 system (Malvern Panalytical, Malvern, UK), while tetraspanins (CD9, CD81, CD63) were analyzed by flow cytometry using the MACSPlex Exosome Kit (Cat. 130-122-209, Miltenyi Biotec) on a MACSQuant^®^ Analyzer 10 (Miltenyi Biotec) to verify the expression of canonical EVsurface markers. Transmission electron microscopy (TEM) of EVs was performed using an Olympus MegaView G2 CCD camera system with the integrated iTEM imaging platform (Olympus, Germany) to further confirm EV morphology and structural characteristics. For functional studies, EVs (50,000 EVs per target cell), labeled with Red Fluorescent Cell Membrane Linker PKH26 (Sigma-Aldrich, St. Louis, MO, USA) to enable visualization of EV uptake by podocytes, were diluted in complete RPMI medium and applied to human immortalized podocytes (hCiPodo; University of Bristol, Bristol, UK) cultured on Superfrost slides in a MACSwell™ Imaging Frame for 24 hours, followed by analysis on the MICS platform. CD63-PKH26 co-staining was used to identify EVs, whereas additional markers, including CD4, were analyzed to investigate their cellular origin.

## 3 Results

### 3.1 Conventional vs. MICS immunohistochemistry

**Figure 1:**
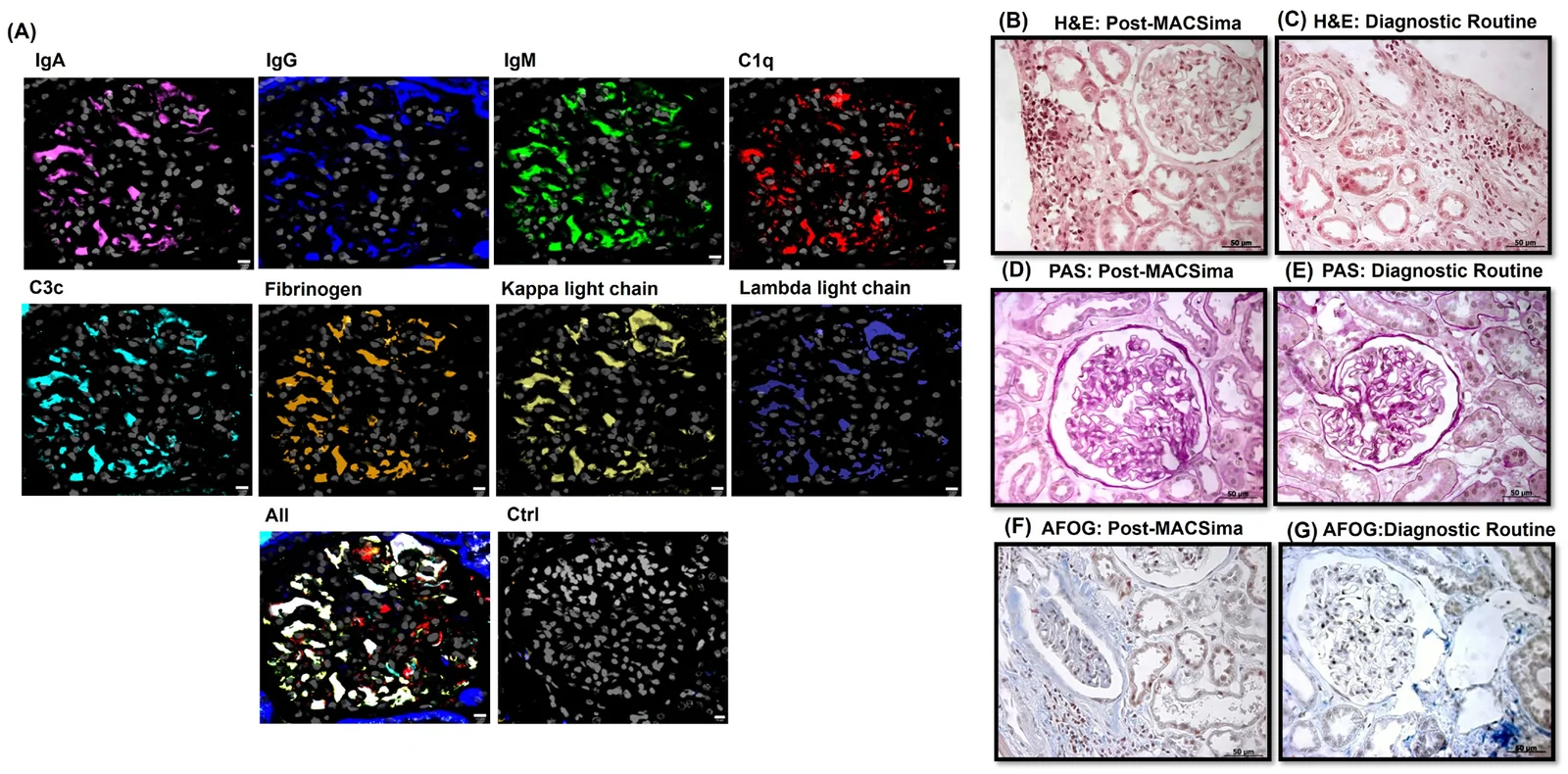
Technical validation of MICS (MACSima Imaging Cyclic Staining) for the reproduction of routine diagnostic immunoprofiles. Multiplex immunofluorescence staining of IgA, IgG, IgM, C1q, C3c, fibrinogen, and κ/λ light chains was performed on a single 3-µm kidney biopsy section from a patient with lupus nephritis (LN), and merge staining in both LN and healthy control kidney biopsy **(A)**. Hematoxylin and Eosin [H&E, **(B, C)**], Periodic Acid–Schiff [PAS, **(D, E)**], and Acid Fuchsin Orange G [AFOG, **(F, G)**] staining of healthy control kidney biopsy sections after the MICS workflow **(B, D, F)** and in routine diagnostic practice **(C, E, G)**. Scale bars: 10 µm and 50 µm.

Given the limited tissue availability from invasive biopsies, we tested whether all diagnostic immunomarkers (IgG, IgA, IgM, C1q, C3c, fibrinogen, κ/λ light chains) could be simultaneously assessed on a single 3-µm tissue section. To validate the ability of MICS to reproduce distinct immunological patterns, we analyzed a native healthy ctrl-KBx and a lupus nephritis kidney biopsy (LN-KBx) with established immunofluorescence staining profiles. MICS faithfully reproduced the characteristic “full-house” immunofluorescence pattern in LN-KBx, absent in the healthy ctrl-KBx, confirming feasibility (**Figure 1A**). We also compared routine stains (PAS, H&E, AFOG) with those performed on ctrl-KBx after MICS. Renal architecture was preserved across methods, with intact structures and no abnormal deposits. Morphology remained comparable (**Figures 1B–G**). MICS allowed simultaneous immunostaining of all diagnostic markers without compromising tissue morphology.

### 3.2 Spatial mapping of kidney structural markers

**Figure 2:**
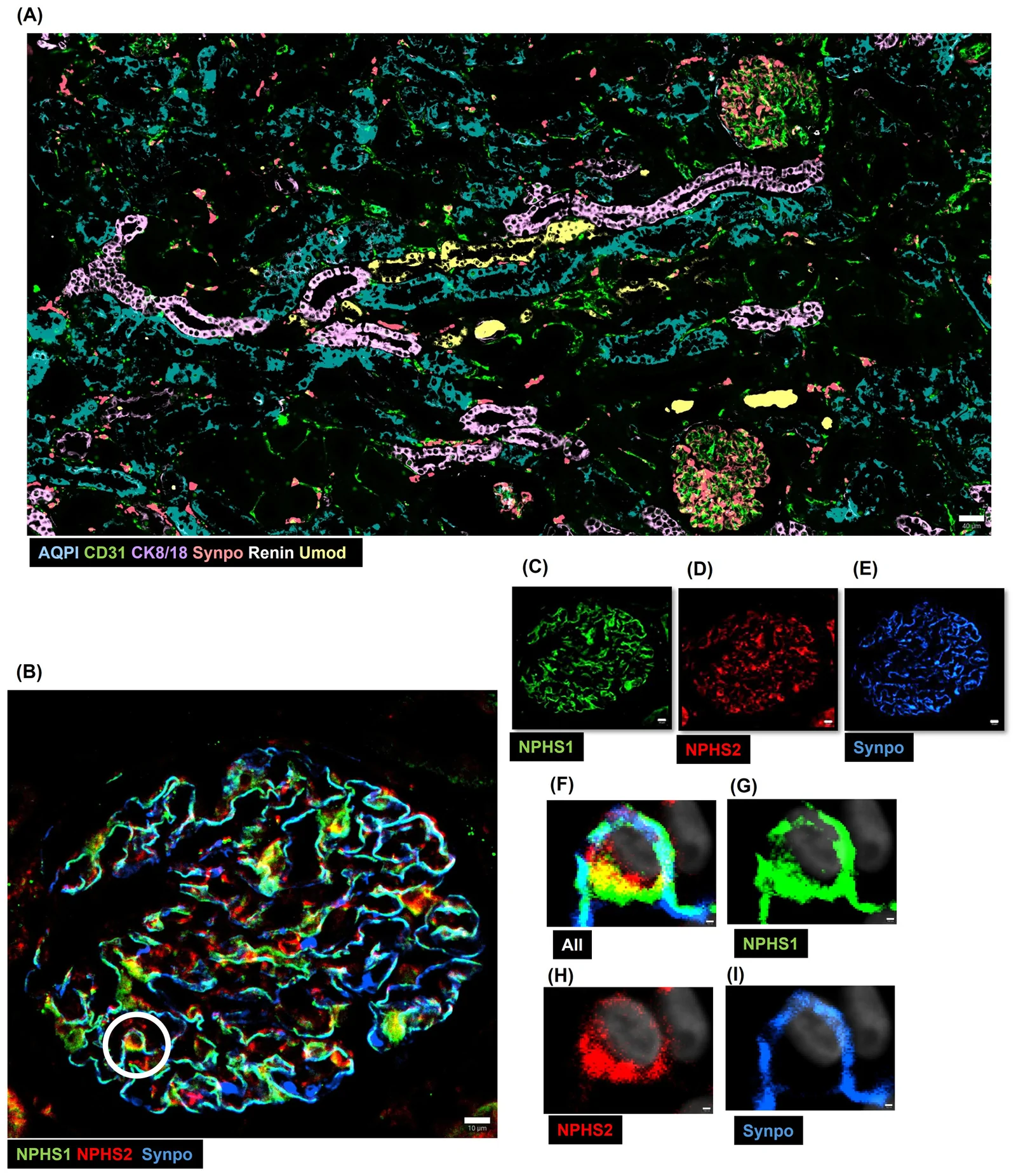
Spatial mapping of renal structural markers. Staining in a healthy control kidney biopsy of Aquaporin-1 (AQP1; proximal tubules), cluster of differentiation 31 (CD31; endothelial cells), cytokeratins 8/18 (CK8/18; distal tubules and collecting ducts), synaptopodin (SYNPO; podocytes), renin (juxtaglomerular apparatus), and uromodulin (UMOD; thick ascending limb) **(A)**. Glomerular staining of nephrin (NPHS1), podocin (NPHS2), and synaptopodin (SYNPO) **(B–E)**, with magnified views highlighting podocyte colocalization **(F–I)**. Scale bars: 40 µm, 10 µm, and 700 nm.

The accurate spatial mapping of kidney markers is essential to interpret tissue organization and function. We then tested key structural markers, namely aquaporin-1 (AQP1), cytokeratin-8/18 (CK8/18), synaptopodin (SYNPO), nephrin/podocin (NPHS1/2), CD31, uromodulin (UMOD), and Renin in healthy ctrl-KBx. As reported in **Figure 2A**, AQP1 labelled proximal tubules, CK8/18 distal-collecting tubules, SYNPO podocytes, CD31 glomerular/peritubular capillaries, UMOD thick ascending limb, and Renin juxtaglomerular cells. NPHS1/2 and SYNPO resulted colocalized in glomeruli, confirming podocytes staining accuracy (**Figures 2B–I****).** MICS can support high-resolution spatial mapping of renal structures, useful for interpreting tissue organization.

### 3.3 Integrating marker localization with functionality and pathway activation

**Figure 3:**
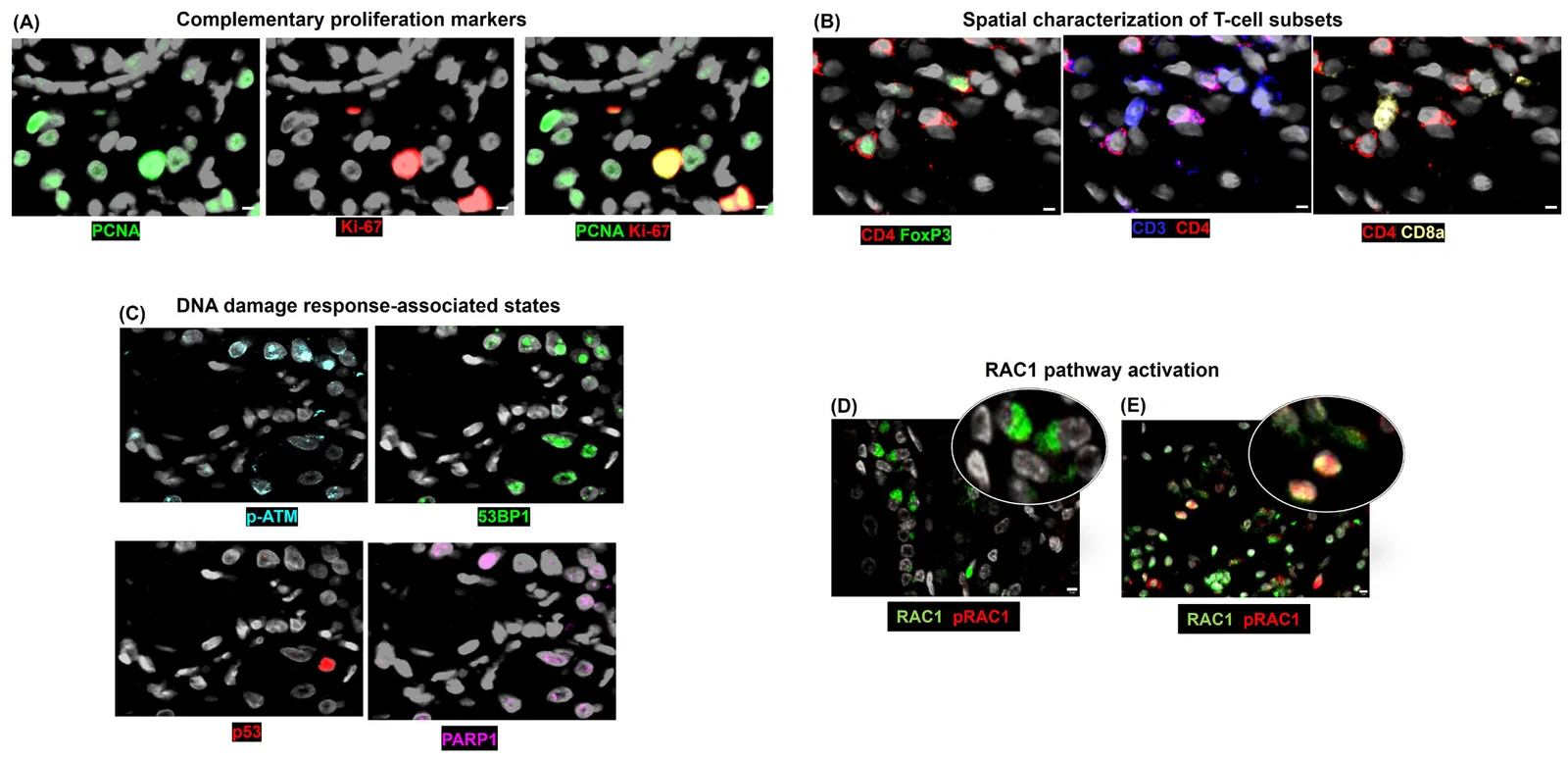
Spatial mapping of proliferation, T-cell subsets, DNA damage response, and RAC1 pathway activation markers. Multiplex imaging in recurrent focal segmental glomerulosclerosis kidney transplant (rFSGS-KTx) biopsies of proliferating cell nuclear antigen (PCNA; marker of DNA replication and cell proliferation) and Ki-67 (marker of cellular proliferation) **(A)**, CD4 (helper T-cell marker), forkhead box P3 (FOXP3; regulatory T-cell transcription factor), CD3 (pan-T-cell marker), and CD8a (cytotoxic T-cell marker) **(B)**, phosphorylated ataxia telangiectasia mutated kinase (p-ATM; marker of DNA damage signaling), p53-binding protein 1 (53BP1; DNA damage response-associated marker), tumor protein p53 (p53; stress response and apoptosis-associated marker), and poly(ADP-ribose) polymerase 1 (PARP1; DNA damage response and repair enzyme) **(C)**, and Ras-related C3 botulinum toxin substrate 1 (RAC1; small GTPase involved in cytoskeletal remodeling and cellular signaling) and phosphorylated RAC1 (p-RAC1; active RAC1-associated form) in control kidney transplant and rFSGS-KTx biopsies **(D, E)**. Scale bars: 5 µm.

To further evaluate the multiplex capability of MICS, we analyzed markers associated with proliferation, immune-cell composition, the DNA damage response, and signaling pathways. The combination of Ki-67 and PCNA highlighted the ability of multiplex imaging to reveal heterogeneous cell-cycle-associated patterns (**Figure 3A**). Immune phenotyping with CD3^+^, CD4^+^, CD8a^+^, and CD4^+^/FOXP3^+^ enabled spatial identification of distinct T-cell populations (pan-T cells, helper T cells, cytotoxic T cells, and regulatory T cells) (**Figure 3B**). The addition of pATM, 53BP1, p53, and PARP1 allowed the visualization of heterogeneous DNA damage-associated profiles (**Figure 3C**). Comparison of RAC1 and p-RAC1 showed reduced RAC1 phosphorylation in the protocol ctrl-KTx biopsy compared with the rFSGS-KTx biopsy, consistent with increased pathway activation in the latter (**Figures 3D, E**).

MICS supported the spatial evaluation of markers associated with cellular processes and signaling-related molecular patterns.

### 3.4 Multiplexed spatial proteomic analysis of ctrl- vs. BKVN-KTx biopsies in the tubule-interstitial compartment and longitudinal evaluation before and after therapy

**Figure 4:**
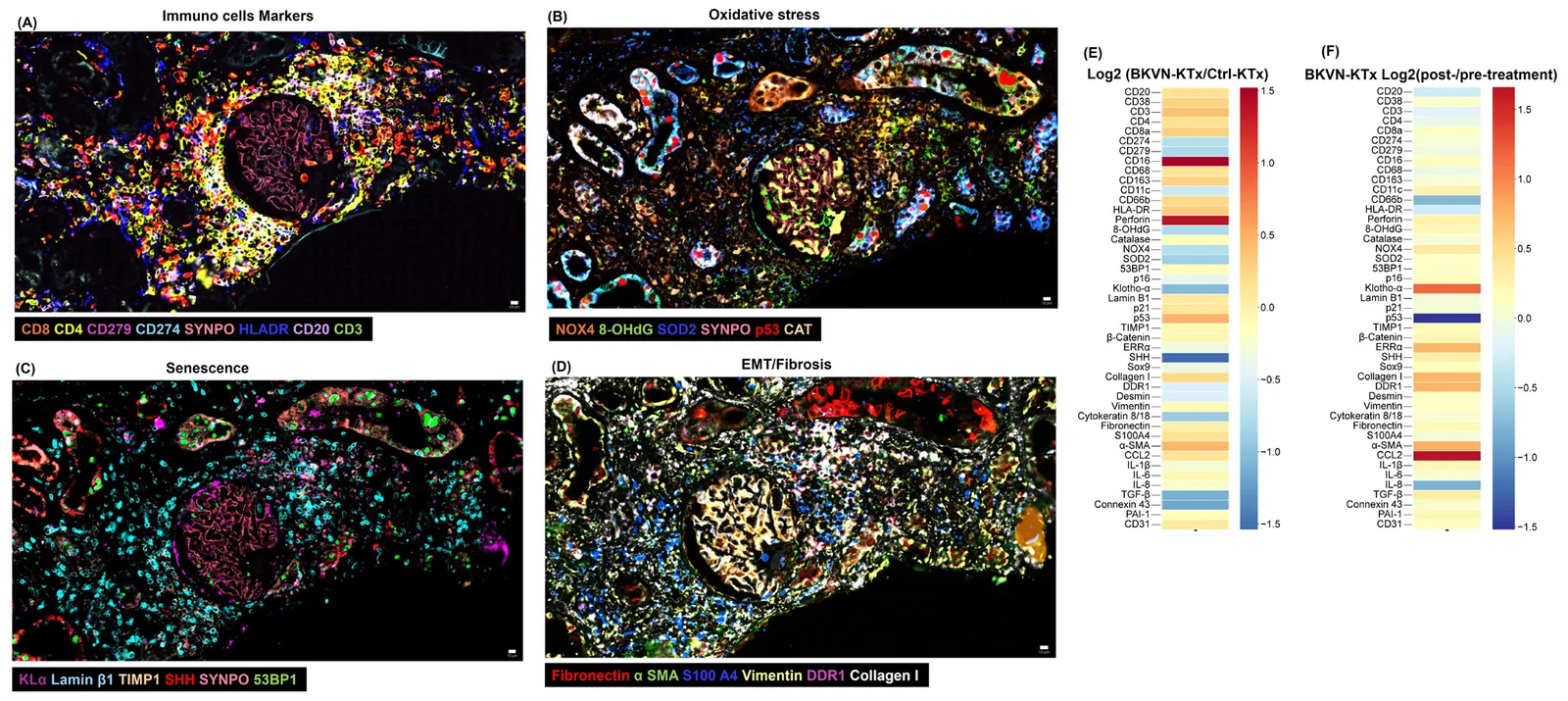
Multiplexed spatial proteomic profiling of BK virus nephropathy in kidney transplant biopsies and longitudinal treatment response. Magnification images of immune, oxidative stress, senescence, and epithelial–mesenchymal transition (EMT)/fibrosis markers in BK virus nephropathy kidney transplant (BKVN-KTx) biopsies **(A–D)**. Heatmaps display the log2 fold change (Log2FC) of selected markers between BKVN-KTx and control kidney transplant (Ctrl-KTx) biopsies (Log2FC = log2[BKVN-KTx/Ctrl-KTx]) and between pre- and post-treatment BKVN-KTx biopsies (Log2FC = log2[post-treatment/pre-treatment]) **(E, F)**. Scale bars: 20 µm.

We generated a 48-marker panel targeting oxidative stress, senescence, EMT/fibrosis, and immune pathways. Representative staining of BKVN-KTx shown in **Figures 4A–D** and Ctrl-KTx biopsies in **Supplementary Figures 1A–D**, respectively. Spatial proteomic profiling was performed in the tubulo-interstitial compartment of control-KTx and BKVN-KTx biopsies. Given the exploratory nature of the analysis, marker modulation was evaluated using log2 fold change (Log2FC; BKVN/Ctrl-KTx) as a descriptive measure rather than formal statistical inference. BKVN-KTx biopsies showed coordinated molecular alterations involving tubular stress responses and selective immune remodeling. Reduced expression of tubular and stress-associated markers was observed, including 8-OHdG (Log2FC −0.79), NOX4 (−0.77), SOD2 (−0.88), CK8/18 (−0.92), Klotho-α (−1.04), and SHH (−1.54). Immune profiling revealed selective modulation of innate and cytotoxic immune signatures, with increased CD16 (+1.52) and Perforin (+1.38), whereas macrophage-associated markers showed only limited changes (CD68 + 0.14; CD163 + 0.27). Adaptive immune markers displayed modest variations, including CD3 (+0.39), CD8a (+0.28), and CD4 (+0.19). Conversely, immune checkpoint markers CD274 (−0.75) and CD279 (−0.80), together with CD11c (−0.63), were reduced, consistent with heterogeneous remodeling of immune compartments rather than generalized immune activation (all details in heatmap **Figure 4E**; **Supplementary Table 3**).

To evaluate treatment-associated changes, we further analyzed a paired pre- and post-treatment BKVN-KTx biopsy from a single patient. Log2 fold change values (post-treatment/pre-treatment) were used as descriptive measures (**Supplementary Table 4**). Following therapy, Klotho-α increased (+1.16) and p53 decreased (−1.52), consistent with modulation of tubular stress and senescence-associated pathways. Conversely, fibrosis-associated markers remained increased, including α-SMA (+0.78), collagen I (+0.73), and DDR1 (+0.74). Immune-related changes were heterogeneous, with increased CCL2 (+1.66) and Perforin (+0.29), whereas CD66b (−0.76) and IL-8 (−0.80) decreased (all details in heatmap **Figure 4F**; **Supplementary Table 4**).

MICS enabled spatial profiling of molecular features associated with disease and treatment.

### 3.5 Distance-based spatial mapping of collagen I-defined fibrotic niches in BKVN-KTx

**Figure 5:**
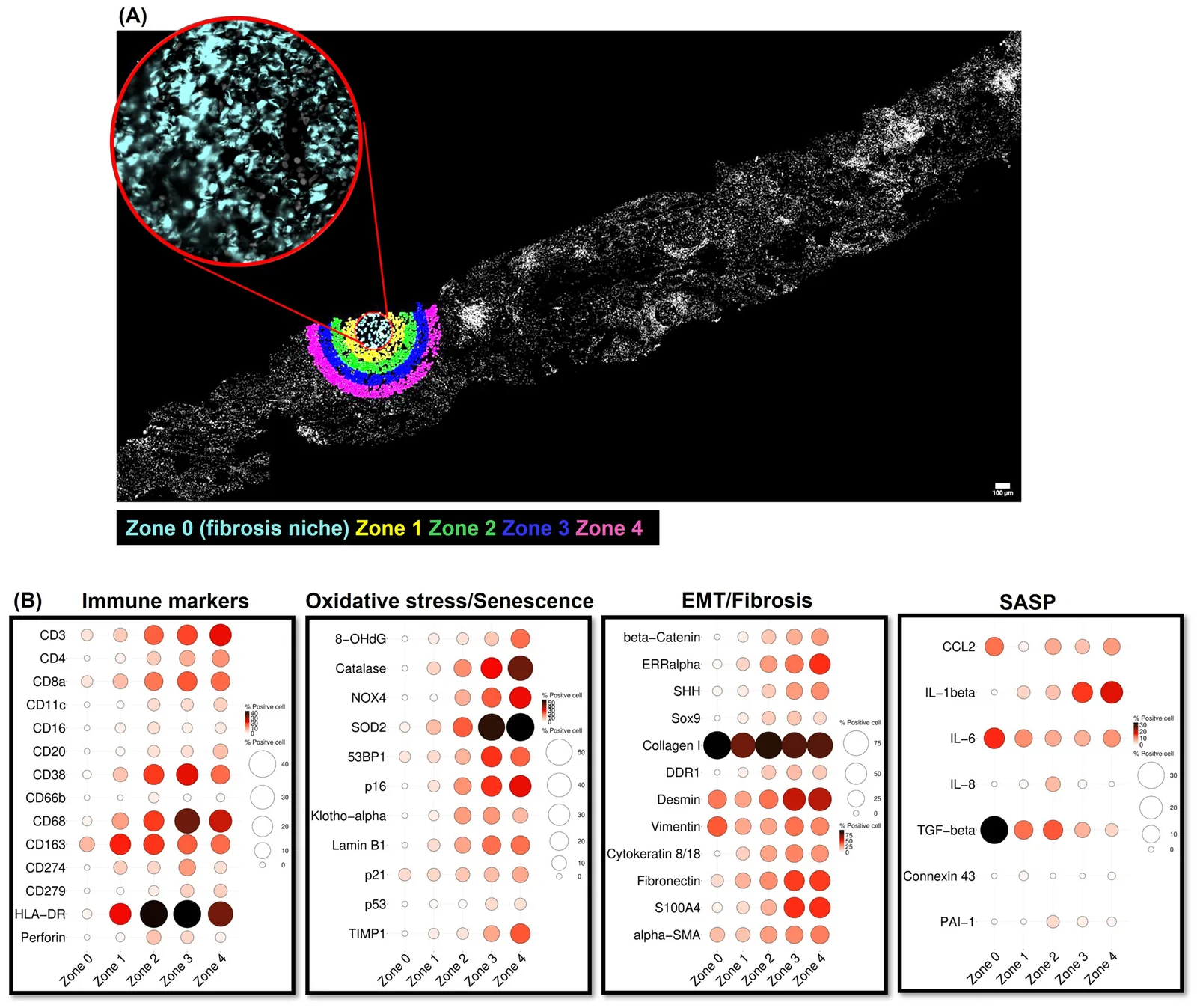
Distance mapping of molecular signatures in fibrosis niches. Distance map from a selected fibrosis niche identified by collagen I (light blue), surrounded by four concentric spatial zones (1–4) representing increasing distances from the fibrosis niche in BK virus nephropathy kidney transplant (BKVN-KTx) **(A)**. Balloon plot showing immune cell, oxidative stress/senescence, epithelial-to-mesenchymal transition (EMT)/fibrosis, and senescence-associated secretory phenotype (SASP) markers across the defined spatial zones (1–4) **(B)**. Scale bar: 100 µm.

We used a distance map from fibrotic niches (zones 0–4) to visualize the spatial distribution of all markers. Fibrotic niches were defined as collagen I-positive areas (zone 0), from which concentric distance zones (zones 1–4) were generated to quantify the distribution of marker-positive cells according to their distance from the fibrotic core (**Figures 5A, B**; **Supplementary Table 5**). MICS may be useful for monitoring spatial changes associated with therapeutic intervention and tissue remodeling.

### 3.6 Spatial proteomic in glomeruli of immune–complement architecture in rFSGS- KTx and LN-Bx

**Figure 6:**
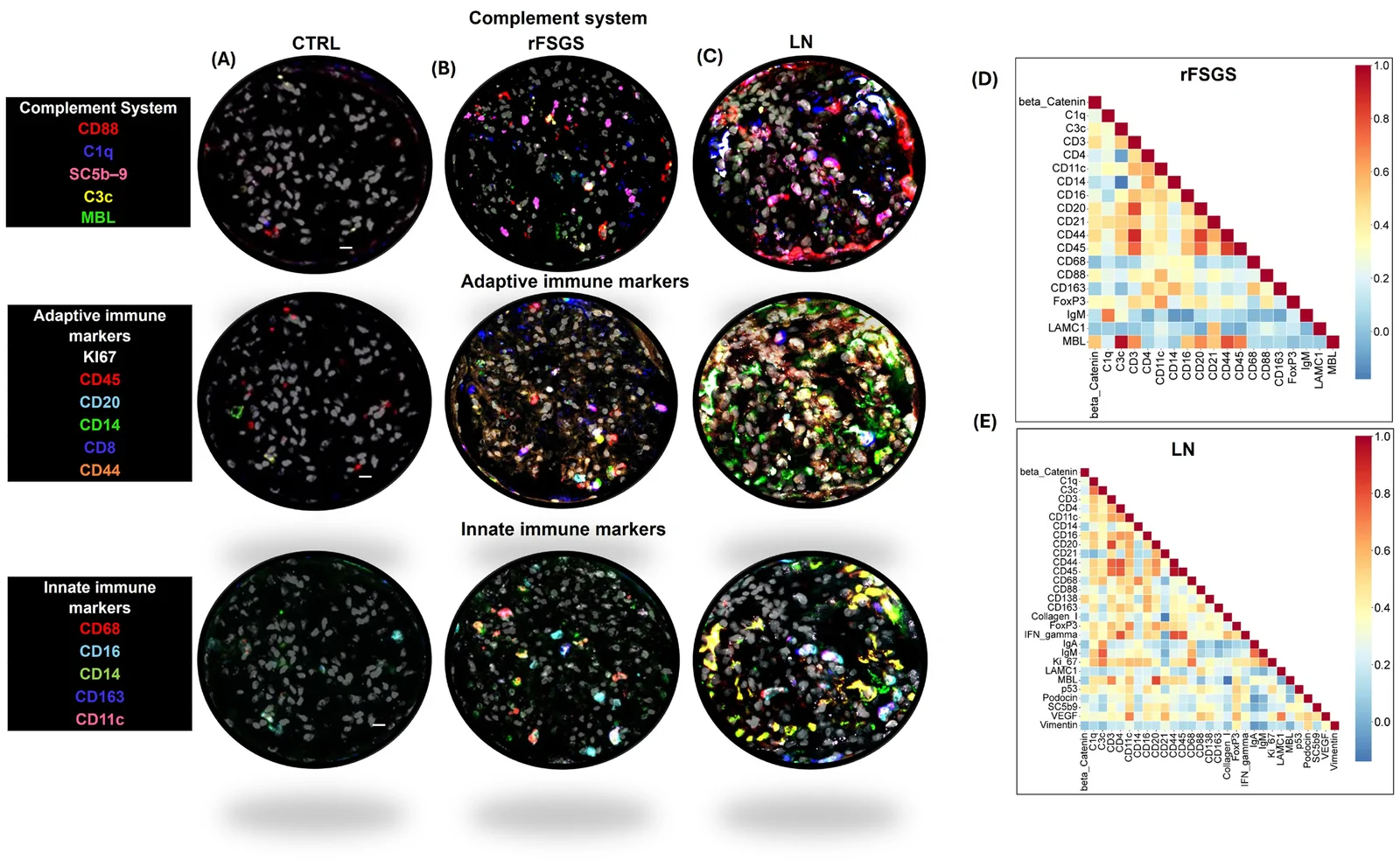
Glomerular immune and complement spatial networks in rFSGS KTx and LN-KBx Multiplex imaging of complement, adaptive, and innate immune markers in the glomeruli of control kidneys, recurrent focal segmental glomerulosclerosis kidney transplant biopsies (rFSGS-KTx) and lupus nephritis kidney biopsies (LN-KBx) **(A–C)**. Spatial correlation heatmaps of immune-complement networks in the glomeruli of rFSGS-KTx and LN-KBx **(D, E)**. Scale bar 10µm.

To explore the glomerular immune panel across kidney diseases, we applied a 30-marker spatial proteomic set focused on innate, and adaptive immunity, and on complement activation. Representative glomerular images are shown for rFSGS-KTx, LN-KBx, and healthy ctrl-KBx and spatial correlation analysis was performed across the full marker panel (**Figures 6A–C**; all data are reported in the repository: https://doi.org/10.6084/m9.figshare.32043915**).** For visualization purposes, the heatmap displays correlations with r>0.50, strong spatial correlations (r ≥ 0.70) were considered, and are discussed in the text (**Figures 6D, E**). In rFSGS-KTx, we identified a coordinated network involving adaptive immune and complement-related markers. C3c showed a strong correlation with MBL (r = 0.95), suggesting a spatial association between different complement activation pathways. CD3 showed strong correlations with CD20 (r = 0.81), CD44 (r = 0.88), and CD45 (r=0.74). CD20 further correlated with CD44 (r = 0.84) and CD45 (r = 0.73), while CD44 correlated with CD45 (r = 0.83) and MBL (r = 0.80). These findings indicate a spatial relationship between immune cell-associated markers and complement activation within rFSGS-KTx glomeruli (**Figure 6D**).

In LN-KBx, spatial correlation analysis identified an immune network involving complement deposition, innate and adaptive immune cells, and proliferative activity. C1q correlated with C3c (r = 0.74), while C3c correlated with IgM (r = 0.78). Adaptive immune markers showed coordinated spatial distribution, with CD3 correlating with CD20 (r = 0.82), CD44 (r = 0.76), and CD45 (r = 0.74). CD4 correlated with IFN-γ (r = 0.80), CD44 (r = 0.80), and CD45 (r = 0.77). Moreover, CD44 showed strong correlations with CD45 (r = 0.95) and IFN-γ (r = 0.80), while CD45 correlated with IFN-γ (r = 0.78). Additional associations included CD20 with MBL (r = 0.82), CD68 with Ki-67 (r = 0.71), IgA with IgM (r = 0.74), and LAMC1 with VEGF (r = 0.71). Overall, the immune network activity and complement deposition are increased and more complex in LN (**Figure 6E**).

MICS supported spatial profiling of molecular patterns in kidney glomerular microenvironments.

### 3.7 Other potential MICS applications: EVs characterization and detection

**Figure 7:**
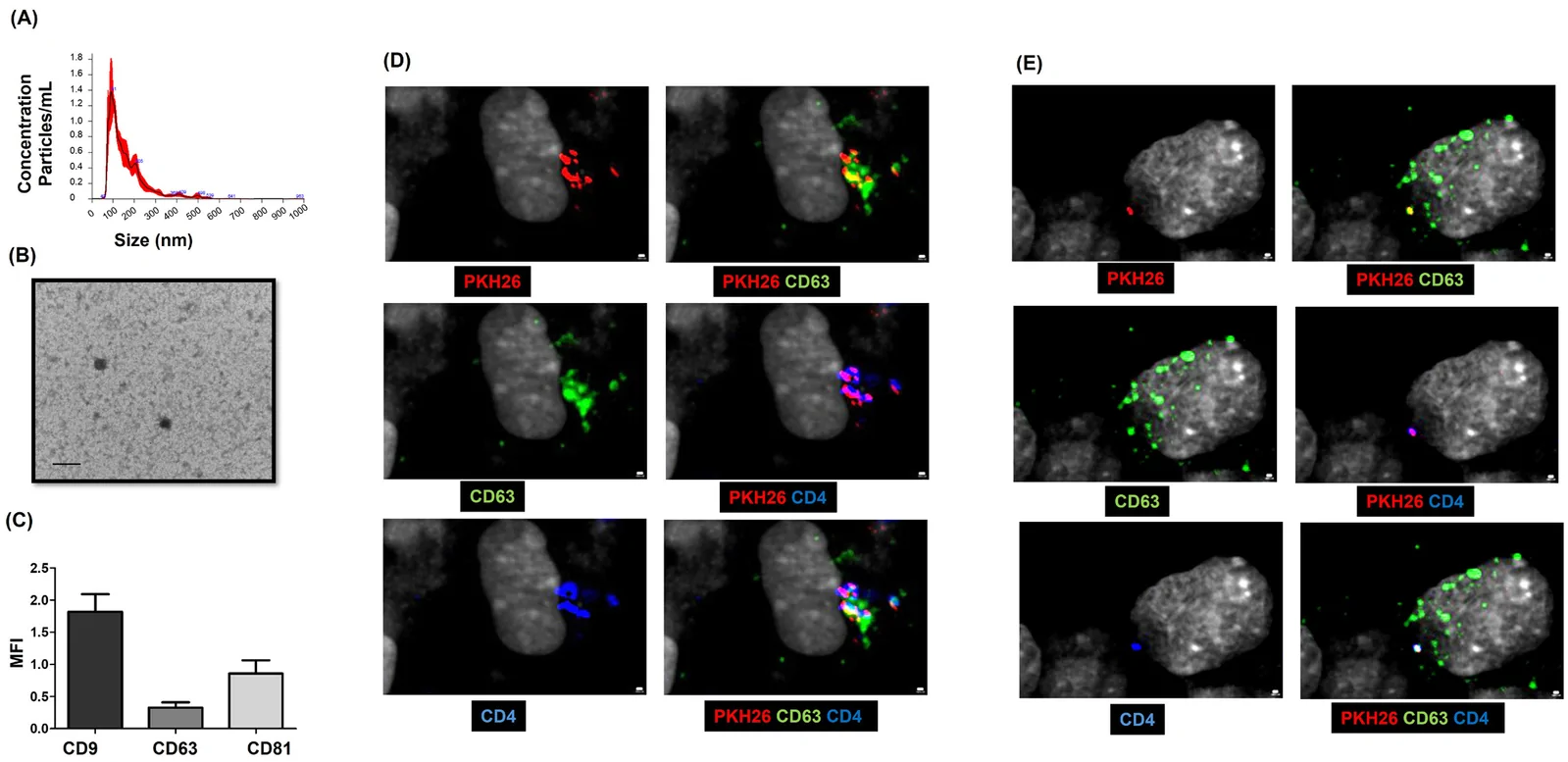
MICS (MACSima Imaging Cyclic Staining)-based extracellular vesicle detection. Size distribution and concentration of extracellular vesicles (EVs) extracted from the plasma of five antibody-mediated rejection kidney transplant patients, analyzed by nanoparticle tracking analysis **(A)**. Morphological quality control of EVs by transmission electron microscopy **(B)**. EV characterization by flow cytometric analysis of CD9, CD63, and CD81 tetraspanins **(C)**. EV uptake by podocytes (nucleus shown in grey) tracked by PKH26 labeling and co-localization with CD63 for EV validation and CD4 for cell origin assessment using MICS imaging **(D, E)**. Scale bars: 500 nm and 10 µm.

To demonstrate potential sub-cellular application of this technology, we isolated EVs from CKD patients and characterized them by Nanoparticle Tracking Analysis (NTA), Transmission Electron Microscopy (TEM), and flow cytometry (FC). NTA showed concentrations up to 1.3×10^9^/mL with mean sizes 161.8 nm, consistent with TEM. FC confirmed the presence of tetraspanins CD9, CD63, and CD81 (**Figures 7A–C****).** As reported in the **Figures 7D, E**, MICS imaging of PKH26-labeled EVs colocalized correctly with CD63 and CD4, supporting the identification of EV-associated structures and suggesting an immune cell-associated phenotype defining a specific cellular origin. MICS extended the spatial analysis of liquid biopsy approaches

## 4 Discussion

In this study, we performed a methodological proof-of-concept study to evaluate the feasibility of a high-plex spatial proteomics workflow for the analysis of routine diagnostic FFPE-KBx specimens, in addition to investigating disease-specific biological differences. By using a single 3 µm biopsy section, we aimed to overcome the need for multiple serial sections required for conventional histopathological and immunohistochemical diagnostic assessment, which can span up to approximately 100 µm of tissue depth. As this was a methodological validation study, the proof-of-concept was independent of whether the biopsy originated from a native or transplanted kidney. The objective was to demonstrate that MICS could support comprehensive renal biopsy diagnostics on a single tissue section in both settings.

To validate compatibility with standard diagnostic workflows, an LN-KBx with a well-established “full-house” immunofluorescence pattern was used as a technical benchmark for multiplex detection of diagnostic immune markers. Multiplex immunofluorescence combined with subsequent conventional histology preserved both signal quality and tissue morphology. This single-section strategy reduces tissue consumption, enables re-staining and panel expansion, minimizes sampling bias, and accelerates processing, with automated acquisition of a 10×2 mm biopsy within 48 hours. Overall, the workflow integrates high-dimensional profiling with conventional histology without compromising tissue integrity.

The limited availability of kidney-specific antibodies and the intrinsic heterogeneity of renal tissue remain important challenges. Nevertheless, antibodies targeting major renal structures performed robustly, with only minor optimization required for a subset of markers. Importantly, the platform supports in-house antibody conjugation, allowing expansion toward less commonly used targets. NPHS1, which is technically challenging due to antigen retrieval constraints, alternative splicing, and weak signal intensity, was reliably detected and partially colocalized with NPHS2 and SYNPO, enabling accurate assessment of podocyte integrity and disease progression consistent with previous evidence indicating differential loss of podocyte marker expression (20, 21).

We further demonstrate that, in addition to high-resolution structural imaging, MICS enables the precise spatial localization of functional markers. The combined assessment of Ki-67 and PCNA, commonly used markers to highlight cell-cycle activity, revealed heterogeneous profiles consistent with distinct proliferative states, underscoring the ability of MICS to distinguish variations associated with different phases of cellular progression. The inclusion of CD3, CD4, CD8a, and FOXP3 enabled a more detailed spatial characterization of T-cell populations, while the combination of 53BP1, pATM, p53, and PARP1 revealed heterogeneous DNA damage-associated profiles, suggesting different components or phases of the cellular stress response. RAC1 and phospho-RAC1 provided a spatial readout of signaling activity associated with cytoskeletal remodeling and podocyte injury responses, the upstream activation of which was previously demonstrated by our group using the MICS system (22). Together, these data demonstrate that MICS can integrate molecular markers to characterize spatially organized cellular responses, with potential relevance for biomarker network discovery.

Moving from technical validation to biological application, we sought to exploit the high-plex capability of MICS to address specific pathophysiological questions, expanding our panel to deeply profile the glomerulus and the tubulointerstitium across two distinct renal pathologies.

Application of a 48-marker panel to ctrl- and BKVN-KTx biopsies revealed coordinated changes in oxidative stress, tubular injury, immune activation, and fibrotic pathways. BKVN-KTx samples showed altered expression of oxidative stress markers (8-OHdG, NOX4, SOD2), decreased tubular markers (CK8/18, Klotho-α), and downregulation of SHH signaling, consistent with impaired repair mechanisms (23, 24). Within the analyzed tubulo-interstitial regions, immune profiling revealed selective modulation of innate, cytotoxic, and adaptive immune signatures. Increased CD16 and Perforin expression suggested enrichment of cytotoxic immune programs involving CD16-positive immune cells and perforin-expressing cytotoxic lymphocytes, whereas limited changes in macrophage-associated markers and reduced CD11c indicated heterogeneous innate immune remodeling rather than generalized inflammation. This pattern is consistent with current evidence that BKVN control depends on the recovery of antiviral cellular immunity through coordinated interactions between innate immune sensing, antigen presentation, and adaptive responses, while maintaining a balance between viral control and alloimmune injury (25, 26).

Longitudinal analysis further indicated partial restoration of protective pathways following therapy, alongside persistent activation of fibrosis-related markers (α-SMA, collagen, DDR1) suggested ongoing extracellular matrix remodeling. Reduced IL-8 and CD66b further indicated modulation of inflammatory and neutrophil-associated activity, potentially reflecting attenuation of SASP-related signaling (27). These findings highlight the potential of the approach to capture spatially resolved molecular dynamics associated with disease progression and therapeutic response.

Importantly, a distance-based framework for analyzing tissue organization, which cannot be achieved using conventional multiplex approaches, was additionally investigated using MICS. Because fibrosis is a major pathological feature of BK virus nephropathy, collagen I-positive fibrotic regions were selected as the reference core for distance mapping, enabling characterization of the spatial organization of the surrounding tissue microenvironment. These findings support the concept that fibrotic regions may represent biologically active microenvironments rather than merely passive end-stage lesions. The same distance-based approach could be readily extended to other tissue or cellular niches, including glomeruli, inflammatory infiltrates, vascular structures, or tumor microenvironments, depending on the biological question (19).

We then extended this spatial proteomic approach to the glomerular compartment in rFSGS-KTx, using LN-KBx as a positive control. In rFSGS-KTx, we observed the presence of multiple complement-related components, including C1q, CD88, MBL, C3c, and C5b-9. While activation of the alternative pathway has been previously reported, the contribution of classical and lectin pathways remains less clearly defined and is still under debate (28–31). Our spatial analysis suggests distinct patterns of immune and complement marker co-occurrence, pointing toward a potentially more restricted immune-complement organization in rFSGS-KTx compared to the broader inflammatory architecture observed in LN-KBx.

These findings should be considered hypothesis-generating, as biological heterogeneity may lead to patient-specific spatial signatures. Larger cohorts will be necessary to determine which spatial relationships are reproducibly associated with disease pathogenesis.

Beyond tissue-based diagnostics, the adaptability of the MICS platform allows it to bridge the gap between solid tissue profiling and circulating biomarkers.

As a proof of this versatility, the additional observation with MICS of EVs pre-stained with PKH26, along with CD63 confirming identity minimizing technical artefacts and CD4 tracing cellular origin, demonstrates its potential for non-invasive liquid biopsy application. Beyond conventional EV characterization methods, the potential value of MICS lies in its ability to analyze individual EVs phenotype, rather than bulk EV populations, within their biological microenvironment, as our case podocytes recipient cells. This capability may be particularly valuable in transplantation, where future applications could enable the simultaneous detection of diagnostic cargo and tissue-specific markers on individual EVs, facilitating the identification of pathogenic subpopulations associated with early graft injury.

A limitation of this study is the small sample size, which was intentionally restricted to enable a detailed methodological evaluation. Accordingly, the results should be interpreted as a proof-of-concept rather than definitive biological conclusions. The correlations and spatial associations described here are intended to illustrate the analytical potential of the platform and to generate hypotheses that will require validation in larger, well-powered patient cohorts. Future studies will be essential to confirm these findings, assess their reproducibility, and determine their clinical relevance across broader transplant populations.

In conclusion, the MICS platform enables highly multiplexed spatial proteomic analysis on minimal tissue input while preserving architecture and allowing downstream analyses. This approach provides integrated molecular profiling across structural, immune, and functional compartments at subcellular resolution, and supports the investigation of spatial and temporal dynamics through longitudinal and distance-based analyses. Extracting high-dimensional molecular information from limited biopsy material, this platform offers a powerful tool for advancing mechanistic and translational research in nephrology, with potential implications for improving disease stratification and guiding targeted therapeutic strategies in solid organ transplantation.

## Data Availability

The original contributions presented in the study are included in the article, in the **Supplementary Material**, and correlation data available in the repository: https://doi.org/10.6084/m9.figshare.32043915.

## Supplementary material

Supplementary Figure 1 **Figure 4**: Multiplexed Spatial Proteomic Profiling of control kidney transplant. Magnification images of immune-, oxidative stress, senescence, and epithelial–mesenchymal transition (EMT)/fibrosis-related markers in control kidney transplant **(A–D)**.

Supplementary Figure 2 MACSima Imaging Cyclic Staining (MICS) of extracellular vesicle detection. Uptake of extracellular vesicles, extracted from the plasma of 5 antibody-mediated rejection kidney transplant patients, in podocytes (nucleus in grey) tracked by PKH26 and co-localized with CD63 and CD4 using MICS imaging **(A–D)**. Scale bar: 10µm.
