# Supplementary material for: Single-section multiplex spatial proteomics of immune microenvironments in kidney transplantation
Source: Front Immunol. 2026 Aug 24;17:1861589. doi: 10.3389/fimmu.2026.1861589 (PMC13547534; doi:10.3389/fimmu.2026.1861589)
Supplement: Supplementary file 1 [file Table1.docx]

Supplementary Material

# 1 Supplementary Tables

# Supplementary Table 1: Summary of patient samples and experimental rational

| **Figure(s)** | **Sample** | **Biopsy type** | **Clinical characteristics** | **Experimental rationale** |
| --- | --- | --- | --- | --- |
| Fig. 1 | LN-KBx | Native kidney biopsy | Lupus nephritis with diagnostic "full-house" immunofluorescence pattern | Technical validation of MICS by comparison with conventional immunopathology and assessment of tissue preservation. |
|  | Healthy ctrl-KBx | Native kidney tissue (nephrectomy specimen) | Histologically normal renal cortex | Healthy reference for tissue preservation assessment. |
| Fig. 2 | Healthy ctrl-KBx | Native kidney tissue (nephrectomy specimen) | Histologically normal renal cortex | High-resolution spatial mapping of normal renal architecture. |
| Fig. 3 | rFSGS-KTx | Kidney transplant biopsy | Recurrent focal segmental glomerulosclerosis | Exploratory spatial assessment of selected cellular processes and pathway-associated markers. |
|  | ctrl-KTx | Kidney transplant biopsy | Protocol biopsy without histological abnormalities | Reference tissue for physiological marker distribution. |
| Figs. 4–5 | BKVN-KTx | Kidney transplant biopsy | BK polyomavirus nephropathy | Characterization of tubulointerstitial inflammation, fibrosis, oxidative stress, senescence, and virus-associated tissue remodeling, including spatial distance mapping relative to the fibrotic niche (Fig. 5). |
|  | Paired BKVN-KTx | Kidney transplant biopsy | Pre-treatment biopsy: BKVN with SV40 positivity; post-treatment biopsy after immunosuppression minimization and IVIG: chronic tubulointerstitial damage with SV40 negativity | Longitudinal assessment of tissue remodeling and immune marker redistribution following therapy. |
|  | ctrl-KTx | Kidney transplant biopsy | Protocol biopsy without histological abnormalities | Healthy transplant reference for tubulointerstitial analyses. |
| Fig. 6 | rFSGS-KTx | Kidney transplant biopsy | Recurrent focal segmental glomerulosclerosis | Glomerulus-focused spatial analysis of innate and adaptive immune pathways, including complement-associated markers. |
|  | LN-KBx | Native kidney biopsy | Lupus nephritis | Reference tissue representing immune-complement activation. |
|  | Healthy ctrl-KBx | Native kidney tissue (nephrectomy specimen) | Histologically normal renal cortex | Baseline reference for physiological glomerular architecture and complement marker distribution. |
| Fig. 7 | Plasma extracellular vesicles (ABMR) | Plasma-derived extracellular vesicles | Active antibody-mediated rejection | Evaluation of MICS spatial resolution at the subcellular level. |

Abbreviations: LN, lupus nephritis; KBx, native kidney biopsy; LN-KBx, native kidney biopsy from a patient with lupus nephritis; ctrl, control; ctrl-KBx, control native kidney biopsy; MICS, MACSima Imaging Cyclic Staining; KTx, kidney transplant; rFSGS, recurrent focal segmental glomerulosclerosis; rFSGS-KTx, kidney transplant biopsy from a patient with recurrent focal segmental glomerulosclerosis; ctrl-KTx, protocol control kidney transplant biopsy; BKVN, BK polyomavirus nephropathy; BKVN-KTx, kidney transplant biopsy from a patient with BK polyomavirus nephropathy; EVs, extracellular vesicles; ABMR, antibody-mediated rejection; SV40, simian virus 40 large T antigen immunostaining; IVIG, intravenous immunoglobulin.

Supplementary Table 2. Functional classification and biological relevance of antibodies used for multiparametric analysis

| **Functional category** | **Vendor** | **Cat.#** | **Antibody** | **Clone** | **Label** | **Biological relevance** |
| --- | --- | --- | --- | --- | --- | --- |
| **DNA damage response and genomic stress** | Abcam | ab214954 | PARP1 | EPR18461 | AF488 | DNA repair enzyme involved in response to DNA strand breaks |
|  | Miltenyi | 130-119-502 | TP53 (p53) | REA1132 | PE | Tumor suppressor involved in stress response, apoptosis and cell-cycle arrest |
|  | Miltenyi | 130-130-617 | 53BP1 | REAL1211 | PE | Marker of DNA double-strand break repair foci |
|  | Novus | NB100-306F | p-ATM (Ser1981) | 10H11.E12 | FITC | Activated ATM marker of DNA damage response signaling |
|  | Santa Cruz | sc-393871 | 8-OHdG | E-8 | PE | Marker of oxidative DNA damage and genomic stress |
| **Cell proliferation,se nescence and cell-cycle regulation** | Abcam | ab233658 | PCNA | PC10 | PE | Marker of DNA replication and proliferating cells |
|  | Miltenyi | 130-120-417 | Ki-67 | REA183 | PE | Marker of active cellular proliferation |
|  | Proteintech | 10355-1-AP | p21/  CDKN1A | Polyclonal | FITC* | Cell-cycle inhibitor associated with stress-induced arrest and senescence |
|  | Novus | NB200-111 | p16/CDKN2A | Polyclonal | PE | Senescence-associated cyclin-dependent kinase inhibitor promoting cell-cycle arrest |
|  | Miltenyi | 130-128-143 | Lamin B1 | REAL1073 | APC | Nuclear structural protein reduced during cellular senescence |
| **Oxidative stress and mitochondrial homeostasis** | Novus | NB110-58849 | NOX4 | Polyclonal | PE | NADPH oxidase involved in reactive oxygen species generation and oxidative signaling |
|  | Novus | NBP2-70342 | Catalase | OTI1B8 | PE | Antioxidant enzyme involved in hydrogen peroxide detoxification |
|  | Proteintech | 24127_1-AP | SOD2 | Polyclonal | PE* | Mitochondrial antioxidant enzyme protecting against oxidative stress |
| **Developmental and regenerative signaling** | Abcam | ab185966 | SOX9 | EPR14335-78 | PE* | Transcription factor regulating progenitor maintenance and differentiation |
|  | Abcam | ab305738 | SHH | EP1190Y | APC | Developmental signaling pathway involved in regeneration and tissue patterning |
| **Cell signaling, migration and cytoskeletal remodeling** | Santa Cruz | sc-514583 | RAC1/2/3 | G-2 | FITC | Rho GTPases involved in cytoskeletal remodeling and cell migration |
|  | Invitrogen | PA5-104640 | p-RAC1 | Polyclonal | APC* | Marker of RAC1 activation and migratory signaling |
| **Inflammatory cytokines and chemokines** | Abcam | ab283818 | IL-1β | RM1009 | FITC* | Pro-inflammatory cytokine involved in innate immune activation and inflammatory signaling |
|  | Santa Cruz | sc-130326 | IL-6 | 1 | PE | Cytokine involved in inflammation and immune activation |
|  | Novus | FAB208A | CXCL8/  IL-8 | 6217 | APC | Chemokine involved in neutrophil recruitment and inflammatory responses |
|  | Novus \| | NBP2-22115 | CCL2/  MCP-1 | 2D8 | PE | Chemokine promoting monocyte/macrophage recruitment and inflammatory cell infiltration |
| Innate immune populations and antigen presentation | Miltenyi | 130-127-908 | CD163 | REA1309 | PE | Marker of macrophage differentiation and alternatively activated macrophage populations |
|  | Miltenyi | 130-128-345 | CD68 | REA1306 | PE | General marker of monocytes/macrophages and tissue-resident macrophage populations |
|  | Miltenyi | 130-128-774 | CD16 | REA1324 | PE | Marker of monocyte/macrophage subsets associated with inflammatory responses |
|  | Miltenyi | 130-128-346 | CD11c | REA1310 | PE | Marker of dendritic cells and activated antigen-presenting myeloid cells |
|  | Miltenyi | 130-117-692 | CD66b | REA306 | APC | Marker of neutrophils and activated granulocytes |
|  | Miltenyi | 130-123-076 | HLA-DR | REAL550 | FITC | Marker of antigen presentation and immune cell activation |
| **Adaptive immune populations** | Miltenyi | 130-120-267 | CD3 | REA1151 | FITC | Pan-T-cell marker identifying total T lymphocyte populations |
|  | Miltenyi | 130-127-906 | CD4 | REA1307 | PE | Marker of helper T lymphocytes involved in immune regulation |
|  | Miltenyi | 130-117-202 | CD8a | REA1024 | APC | Marker of cytotoxic T lymphocytes involved in immune-mediated cytotoxicity |
|  | Abcam | ab237888 | Perforin | PRF1/2468 | APC* | Cytotoxic effector molecule of NK cells and cytotoxic T lymphocytes |
|  | Miltenyi | 130-118-292 | CD20 | REA1087 | FITC | Marker of B-cell lineage and mature B-cell populations |
|  | Miltenyi | 130-127-808 | FoxP3 | REA1253 | PE | Transcription factor defining regulatory T-cell populations |
| **Immune checkpoint and immune regulation** | Miltenyi | 130-127-907 | CD274/  PD-L1 | REA1308 | PE | Immune checkpoint ligand involved in regulation of T-cell responses |
|  | Miltenyi | 130-120-383 | CD279/  PD-1 | REA1165 | APC | Inhibitory receptor associated with T-cell exhaustion and immune regulation |
|  | Miltenyi | 130-126-438 | CD38 | REAL719 | PE | Immune activation marker involved in cellular activation and NAD+ metabolism |
| **Complement activation and humoral immunity** | DAKO | F0201 | C3c | Polyclonal | FITC | Marker of complement activation and tissue complement deposition |
|  | DAKO | F0254 | C1q | Polyclonal | FITC | Initiating component of the classical complement pathway |
|  | DAKO | F0202 | IgG | Polyclonal | FITC | Immunoglobulin involved in adaptive humoral immune responses |
|  | DAKO | F0203 | IgM | Polyclonal | FITC | Early immunoglobulin response marker during immune activation |
|  | DAKO | F0204 | IgA | Polyclonal | FITC | Immunoglobulin involved in mucosal and humoral immunity |
|  | DAKO | F0198 | Kappa Light Chains | Polyclonal | FITC | Immunoglobulin light-chain marker reflecting B-cell and plasma-cell activity |
|  | DAKO | F0199 | Lambda Light Chains | Polyclonal | FITC | Immunoglobulin light-chain marker reflecting B-cell and plasma-cell activity |
| **Coagulation and inflammatory remodeling** | DAKO | F0111 | Fibrinogen | Polyclonal | FITC | Coagulation protein involved in inflammation and tissue repair |
| **Extracellular matrix remodeling and fibrosis** | Abcam | ab211926 | TIMP1 | EPR18352 | FITC* | Matrix metalloproteinase inhibitor involved in extracellular matrix turnover and fibrotic remodeling |
|  | Miltenyi | 130-127-017 | Collagen I | REAL958 | APC | Major extracellular matrix protein increased during fibrosis and tissue remodeling |
|  | Novus | NBP1-91258F | Fibronectin | Polyclonal | FITC | ECM glycoprotein involved in fibrosis and tissue remodeling |
|  | Santa Cruz | sc-130348 | TGF-β1 | 3C11 | PE | Master regulator of fibrotic responses and extracellular matrix deposition |
|  | Santa Cruz | sc-5297 | PAI-1/SERPINE1 | C-9 | PE | Fibrosis- and senescence-associated regulator of extracellular matrix turnover |
|  | Novus | NB300-978 | ACTA2  (α-SMA) | Polyclonal | FITC* | Marker of activated myofibroblasts and fibrotic activation |
| **Mesenchymal and stromal phenotype** | Miltenyi | 130-118-361 | Vimentin | REA409 | APC | Mesenchymal marker of stromal activation and epithelial-to-mesenchymal transition |
|  | Miltenyi | 130-134-196 | S100A4 | REAL1572 | FITC | Marker associated with activated fibroblasts, cell migration and extracellular matrix remodeling |
|  | Proteintech | 10536-1-AP | DDR1 | Polyclonal | FITC* | Collagen receptor involved in extracellular matrix sensing, cellular adhesion and fibrotic signaling |
|  | Miltenyi | 130-119-491 | Desmin | REA1134 | APC | Intermediate filament marker associated with mesenchymal and muscle cell phenotypes |
| **Epithelial phenotype and tissue architecture** | Miltenyi | 130-131-849 | Cytokeratin 8/18 | REAL1358 | PE | Marker of epithelial cell identity, differentiation and tissue architecture maintenance |
|  | Miltenyi | 130-135-494 | CD324/E-cadherin | REA1319 | APC | Cell adhesion molecule maintaining epithelial integrity, polarity and intercellular junctions |
|  | Miltenyi | 130-123-546 | β-catenin | REA480 | FITC | Protein involved in cell adhesion and Wnt signaling regulation |
| **Endothelial phenotype and vascular function** | Miltenyi | 130-128-769 | CD31/  PECAM-1 | REA1312 | PE | Endothelial cell marker used for vascular identification and angiogenesis assessment |
|  | Santa Cruz | sc-9989 | VE-cadherin | F-8 | FITC | Endothelial-specific adhesion molecule involved in vascular integrity and endothelial junction maintenance |
| **Endothelial and epithelial transport functions** | Santa Cruz | sc-32737 | AQP1 | gen-22 | FITC | Water channel expressed in endothelial and epithelial compartments involved in fluid transport |
| **Cell communication and tissue organization** | Miltenyi | 130-127-403 | Connexin 43 (GJA1) | REAL935 | PE | Gap junction protein involved in intercellular communication and tissue organization |
| **Renal function and tubular epithelial markers** | Abcam | ab225743 | Renin | EPR20693 | APC* | Component of the renin–angiotensin system involved in renal homeostasis and blood pressure regulation |
|  | Santa Cruz | sc-271022 | Uromodulin (THP) | B-2 | FITC | Marker of tubular epithelial function and kidney homeostasis |
| **Renal function and podocyte markers** | Novus | NBP1-77303F | Nephrin | Polyclonal | FITC | Podocyte slit diaphragm protein essential for maintenance of the glomerular filtration barrier |
|  | Sigma-Aldrich | P0372 | Podocin | Polyclonal | APC* | Podocyte structural protein involved in slit diaphragm organization and filtration barrier integrity |
|  | Proteintech | CL594-67339 | Synaptopodin | 1E1B1 | PE | Podocyte cytoskeletal protein involved in filtration barrier maintenance and podocyte stability |
| **Renal aging-related pathways and homeostasis** | Proteintech | 28100-1-AP | Klotho | N/A | APC* | Protein involved in kidney homeostasis, mineral metabolism and aging-associated pathways |
| **Extracellular vesicles and cellular communication** | Miltenyi | 130-108-813 | CD63 (MACSPlex EV Kit IO, human) | H5C6 | APC | Tetraspanin associated with extracellular vesicles, exosomes and endosomal compartments |
| **Metabolic regulation and mitochondrial function** | Novus | NBP1-47254F | ERRα/NR3B1 | Polyclonal | FITC | Nuclear receptor regulating mitochondrial metabolism, oxidative capacity and energy homeostasis |
|  |  |  |  |  |  |  |

*Conjugated with Kit (FITC/PE/APC) **Abbreviations:**AF488, Alexa Fluor 488; APC, allophycocyanin; ACTA2, actin alpha 2 (α-smooth muscle actin); ATM, ataxia telangiectasia mutated; CCL2/MCP-1, C-C motif chemokine ligand 2/monocyte chemoattractant protein-1; CD, cluster of differentiation; CDKN1A, cyclin-dependent kinase inhibitor 1A; CDKN2A, cyclin-dependent kinase inhibitor 2A; CCL, C-C motif chemokine ligand; CXCL8/IL-8, C-X-C motif chemokine ligand 8/interleukin-8; DDR1, discoidin domain receptor 1; ECM, extracellular matrix; EV, extracellular vesicle; FITC, fluorescein isothiocyanate; FoxP3, forkhead box P3; GJA1, gap junction alpha-1 protein (connexin 43); HLA-DR, human leukocyte antigen-DR; Ig, immunoglobulin; IL, interleukin; KL, Klotho; NOX4, NADPH oxidase 4; NPHS1, nephrin; NPHS2, podocin; APC, allophycocyanin; PAI-1, plasminogen activator inhibitor-1; PARP1, poly(ADP-ribose) polymerase 1; PCNA, proliferating cell nuclear antigen; PD-1, programmed cell death protein 1; PD-L1, programmed death-ligand 1; PE, phycoerythrin; RAC1, Ras-related C3 botulinum toxin substrate 1; SHH, Sonic Hedgehog; SOD2, superoxide dismutase 2; SOX9, SRY-box transcription factor 9; SYNPO, synaptopodin; TGF-β, transforming growth factor beta; THP/UMOD, Tamm-Horsfall protein/uromodulin; TIMP1, tissue inhibitor of metalloproteinases 1; TP53, tumor protein p53; 53BP1, p53-binding protein 1; VEGF, vascular endothelial growth factor; β-catenin, beta-catenin.

**Supplementary Table 3.** Mean normalized marker expression and log2 fold change analysis in BKVN and control kidney transplant samples.

| **Category** | **Marker** | **Ctrl-Tx (N=3)** | **BKVN-KTx (N=3)** | **Log2FC (BKVN/CtrlTx)** |
| --- | --- | --- | --- | --- |
| **Immune markers** | CD20 | 1597.7 | 1782.5 | 0.16 |
| **Immune markers** | CD38 | 536.9 | 645.7 | 0.27 |
| **Immune markers** | CD3 | 1113.2 | 1455.0 | 0.39 |
| **Immune markers** | CD4 | 1048.2 | 1192.0 | 0.19 |
| **Immune markers** | CD8a | 1083.1 | 1314.3 | 0.28 |
| **Immune markers** | CD274 | 636.4 | 377.2 | -0.75 |
| **Immune markers** | CD279 | 62.0 | 35.7 | -0.80 |
| **Immune markers** | CD16 | 158.5 | 454.1 | 1.52 |
| **Immune markers** | CD68 | 764.1 | 843.0 | 0.14 |
| **Immune markers** | CD163 | 1339.1 | 1616.3 | 0.27 |
| **Immune markers** | CD11c | 1018.5 | 657.0 | -0.63 |
| **Immune markers** | CD66b | 92.5 | 108.3 | 0.23 |
| **Immune markers** | HLA-DR | 5556.2 | 6774.1 | 0.29 |
| **Immune markers** | Perforin | 294 | 766 | 1.38 |
| **Oxidative Stress** | 8-OHdG | 633.6 | 365.9 | -0.79 |
| **Oxidative Stress** | Catalase | 7195.1 | 6510.6 | -0.14 |
| **Oxidative Stress** | NOX4 | 774.1 | 454.9 | -0.77 |
| **Oxidative Stress** | SOD2 | 4611.4 | 2507.1 | -0.88 |
| **Senescence** | 53BP1 | 714.4 | 665.4 | -0.10 |
| **Senescence** | p16 | 755.2 | 572.1 | -0.40 |
| **Senescence** | Klotho-α | 881.5 | 429.7 | -1.04 |
| **Senescence** | Lamin B1 | 1574.3 | 1655.6 | 0.07 |
| **Senescence** | p21 | 886.7 | 956.1 | 0.11 |
| **Senescence** | p53 | 194.7 | 273.2 | 0.49 |
| **Senescence** | TIMP1 | 1675.0 | 1593.0 | -0.07 |
| **EMT triggering factors** | β-Catenin | 1212.7 | 1171.8 | -0.05 |
| **EMT triggering factors** | ERRα | 1399.9 | 1127.8 | -0.31 |
| **EMT triggering factors** | SHH | 4945.8 | 1701.9 | -1.54 |
| **EMT triggering factors** | Sox9 | 701.9 | 545.7 | -0.36 |
| **EMT/Fibrosis** | Collagen I | 423.1 | 494.5 | 0.22 |
| **EMT/Fibrosis** | DDR1 | 1707 | 1196.7 | -0.51 |
| **EMT/Fibrosis** | Desmin | 1840.1 | 1287.6 | -0.52 |
| **EMT/Fibrosis** | Vimentin | 1196.5 | 1133.3 | -0.08 |
| **EMT/Fibrosis** | Cytokeratin 8/18 | 608.8 | 322.4 | -0.92 |
| **EMT/Fibrosis** | Fibronectin | 1700.7 | 1717.6 | 0.01 |
| **EMT/Fibrosis** | S100A4 | 5712.2 | 6436.2 | 0.17 |
| **EMT/Fibrosis** | α-SMA | 916.4 | 1281.5 | 0.48 |
| **SASP** | CCL2 | 2420.8 | 2665.3 | 0.14 |
| **SASP** | IL-1β | 595.5 | 495.4 | -0.27 |
| **SASP** | IL-6 | 649 | 615.6 | -0.08 |
| **SASP** | IL-8 | 178.4 | 154.6 | -0.21 |
| **SASP** | TGF-β | 212.8 | 98.2 | -1.12 |
| **SASP** | Connexin 43 | 374.4 | 167.6 | -1.16 |
| **SASP** | PAI-1 | 617 | 575.8 | -0.10 |
| **Structural Markers** | CD31 | 703.9 | 742.3 | 0.08 |

Log2 fold change was calculated as log2 (mean BKVN-KTx/mean Ctrl-KTx). Given the exploratory nature of this Technical Note and the limited sample size (n=3 per group), Log2FC values are presented as descriptive and hypothesis-generating measures and were not used for statistical inference. **Abbreviations:** BKVN, BK virus nephropathy; KTx, kidney transplant; Ctrl, control; EMT, epithelial-to-mesenchymal transition; SASP, senescence-associated secretory phenotype; CD, cluster of differentiation; HLA-DR, human leukocyte antigen-DR; 8-OHdG, 8-hydroxy-2′-deoxyguanosine; NOX4, NADPH oxidase 4; SOD2, superoxide dismutase 2; 53BP1, p53-binding protein 1; p16, cyclin-dependent kinase inhibitor 2A; p21, cyclin-dependent kinase inhibitor 1A; p53, tumor protein p53; TIMP1, tissue inhibitor of metalloproteinases 1; ERRα, estrogen-related receptor alpha; SHH, sonic hedgehog; DDR1, discoidin domain receptor 1; α-SMA, alpha-smooth muscle actin; CCL2, C-C motif chemokine ligand 2; IL, interleukin; TGF-β, transforming growth factor beta; PAI-1, plasminogen activator inhibitor-1.

**Supplementary Table 4.** Mean normalized marker expression and log2 change analysis in paired pre- and post-treatment BKVN kidney transplant biopsies

| **Category** | **Marker** | **Pre-treatment** | **Post-treatment** | **Log2FC (Post/Pre)** |
| --- | --- | --- | --- | --- |
| **Immune markers** | CD20 | 2258.1 | 1829.7 | -0.30 |
| **Immune markers** | CD38 | 683.5 | 716.8 | 0.07 |
| **Immune markers** | CD3 | 1974.2 | 1728.1 | -0.19 |
| **Immune markers** | CD4 | 1339.5 | 1263.8 | -0.08 |
| **Immune markers** | CD8a | 1670.3 | 1799.9 | 0.11 |
| **Immune markers** | CD274 | 249.2 | 254.8 | 0.03 |
| **Immune markers** | CD279 | 40.7 | 39.9 | -0.03 |
| **Immune markers** | CD16 | 400.1 | 458.8 | 0.20 |
| **Immune markers** | CD68 | 887.4 | 853.9 | -0.06 |
| **Immune markers** | CD163 | 1847.1 | 1882.0 | 0.03 |
| **Immune markers** | CD11c | 647.3 | 782.1 | 0.27 |
| **Immune markers** | CD66b | 159.1 | 94.1 | -0.76 |
| **Immune markers** | HLA-DR | 8404.7 | 6848.2 | -0.30 |
| **Immune markers** | Perforin | 232.5 | 284.1 | 0.29 |
| **Oxidative Stress** | 8-OHdG | 550.5 | 637.7 | 0.21 |
| **Oxidative Stress** | Catalase | 6575.1 | 6636.6 | 0.01 |
| **Oxidative Stress** | NOX4 | 709.8 | 902.7 | 0.35 |
| **Oxidative Stress** | SOD2 | 3884.9 | 4158.8 | 0.10 |
| **Senescence** | 53BP1 | 688.5 | 733.7 | 0.09 |
| **Senescence** | p16 | 462.6 | 526.9 | 0.19 |
| **Senescence** | Klotho-α | 570.5 | 1271.5 | 1.16 |
| **Senescence** | Lamin B1 | 2126.7 | 2141.4 | 0.01 |
| **Senescence** | p21 | 862.2 | 870.8 | 0.01 |
| **Senescence** | p53 | 485.9 | 169.3 | -1.52 |
| **Senescence** | TIMP1 | 1658.0 | 1894.3 | 0.19 |
| **EMT triggering factors** | β-Catenin | 1167.4 | 1376.2 | 0.24 |
| **EMT triggering factors** | ERRα | 1339.9 | 2241.6 | 0.74 |
| **EMT triggering factors** | SHH | 2523.0 | 3119.7 | 0.31 |
| **EMT triggering factors** | Sox9 | 537.1 | 609.4 | 0.18 |
| **EMT/Fibrosis** | Collagen I | 456.4 | 757.4 | 0.73 |
| **EMT/Fibrosis** | DDR1 | 1339.9 | 2241.6 | 0.74 |
| **EMT/Fibrosis** | Desmin | 1291.0 | 1406.8 | 0.12 |
| **EMT/Fibrosis** | Vimentin | 1217.7 | 1329.6 | 0.13 |
| **EMT/Fibrosis** | Cytokeratin 8/18 | 490.6 | 507.1 | 0.05 |
| **EMT/Fibrosis** | Fibronectin | 1718.6 | 2017.0 | 0.23 |
| **EMT/Fibrosis** | S100A4 | 6793.1 | 6895.5 | 0.02 |
| **EMT/Fibrosis** | α-SMA | 933.5 | 1607.7 | 0.78 |
| **SASP** | CCL2 | 2891.1 | 9145.5 | 1.66 |
| **SASP** | IL-1β | 497.3 | 587.5 | 0.24 |
| **SASP** | IL-6 | 612.4 | 625.5 | 0.03 |
| **SASP** | IL-8 | 308.1 | 176.8 | -0.80 |
| **SASP** | TGF-β | 80.7 | 101.0 | 0.32 |
| **SASP** | Connexin 43 | 158.3 | 164.6 | 0.06 |
| **SASP** | PAI-1 | 216.0 | 254.6 | 0.24 |
| **Structural Markers** | CD31 | 829.8 | 907.0 | 0.13 |

Log2FC was calculated as log2(mean post-treatment BKVN-KTx / mean pre-treatment BKVN-KTx). Given the exploratory nature of this longitudinal analysis and the single paired case, Log2FC values are presented as descriptive and hypothesis-generating measures and were not used for statistical inference. Abbreviations: BKVN, BK virus nephropathy; KTx, kidney transplant; EMT, epithelial-to-mesenchymal transition; SASP, senescence-associated secretory phenotype; CD, cluster of differentiation; HLA-DR, human leukocyte antigen-DR; 8-OHdG, 8-hydroxy-2′-deoxyguanosine; NOX4, NADPH oxidase 4; SOD2, superoxide dismutase 2; 53BP1, p53-binding protein 1; p16, cyclin-dependent kinase inhibitor 2A; p21, cyclin-dependent kinase inhibitor 1A; p53, tumor protein p53; TIMP1, tissue inhibitor of metalloproteinases 1; ERRα, estrogen-related receptor alpha; SHH, sonic hedgehog; DDR1, discoidin domain receptor 1; α-SMA, alpha-smooth muscle actin; CCL2, C-C motif chemokine ligand 2; IL, interleukin; TGF-β, transforming growth factor beta; PAI-1, plasminogen activator inhibitor-1.

Supplementary Table 5. Spatial distribution of marker-positive cells within distance–map–derived zone

|  |  | **Zone 0 (%)** | **Zone 1 (%)** | **Zone 2 (%)** | **Zone 3 (%)** | **Zone 4 (%)** |
| --- | --- | --- | --- | --- | --- | --- |
| **Immune Markers** | CD3 | 3.2 | 6.0 | 17.1 | 19.4 | 23.7 |
|  | CD4 | 0.0 | 1.7 | 6.0 | 9.4 | 12.4 |
|  | CD8a | 3.2 | 7.7 | 15.1 | 18.1 | 16.4 |
|  | CD11c | 0.0 | 0.3 | 4.0 | 4.0 | 5.4 |
|  | CD16 | 0.0 | 2.0 | 3.7 | 2.0 | 2.7 |
|  | CD20 | 0.0 | 1.0 | 3.7 | 3.7 | 7.4 |
|  | CD38 | 0.8 | 7.0 | 20.1 | 24.1 | 16.4 |
|  | CD66b | 0.0 | 0.0 | 2.3 | 0.3 | 0.7 |
|  | CD68 | 1.6 | 11.0 | 20.1 | 34.4 | 26.4 |
|  | CD163 | 8.0 | 21.4 | 20.4 | 17.4 | 16.4 |
|  | CD274 | 0.0 | 5.7 | 4.7 | 11.4 | 4.0 |
|  | CD279 | 0.0 | 0.3 | 2.3 | 5.4 | 5.4 |
|  | HLA-DR | 1.6 | 23.1 | 43.1 | 44.1 | 33.8 |
|  | Perforin | 0.0 | 1.0 | 7.0 | 4.7 | 2.0 |
| **Oxidative Stress/ Senescence** | 8-OHdG | 0.0 | 3.3 | 4.7 | 8.4 | 21.4 |
|  | Catalase | 0.0 | 6.7 | 17.1 | 30.1 | 45.5 |
|  | NOX4 | 0.0 | 0.3 | 12.4 | 23.4 | 31.1 |
|  | SOD2 | 2.4 | 10.0 | 23.4 | 53.8 | 58.5 |
|  | 53BP1 | 4.0 | 5.7 | 11.4 | 27.4 | 22.7 |
|  | p16 | 0.0 | 4.7 | 16.4 | 27.4 | 31.4 |
|  | Klotho-α | 0.0 | 2.7 | 15.1 | 15.7 | 10.7 |
|  | Lamin B1 | 0.0 | 5.4 | 13.7 | 21.1 | 21.1 |
|  | p21 | 5.6 | 6.0 | 8.7 | 10.0 | 13.0 |
|  | p53 | 0.0 | 1.0 | 1.0 | 5.7 | 4.7 |
|  | TIMP1 | 0.0 | 3.0 | 4.3 | 13.4 | 24.1 |
| **EMT/Fibrosis** | β-Catenin | 0.0 | 4.7 | 14.7 | 20.7 | 25.8 |
|  | ERRα | 2.4 | 11.0 | 25.8 | 34.4 | 46.5 |
|  | SHH | 3.2 | 5.4 | 13.4 | 25.4 | 28.8 |
|  | Sox9 | 0.0 | 5.0 | 12.7 | 14.7 | 10.0 |
|  | Collagen I | 98.4 | 75.9 | 91.3 | 81.9 | 81.3 |
|  | DDR1 | 0.0 | 4.0 | 14.4 | 17.4 | 14.0 |
|  | Desmin | 33.6 | 23.7 | 35.1 | 59.5 | 62.2 |
|  | Vimentin | 40.0 | 22.7 | 23.7 | 35.1 | 30.4 |
|  | Cytokeratin 8/18 | 0.0 | 12.0 | 23.4 | 30.4 | 27.4 |
|  | Fibronectin | 9.6 | 19.7 | 28.8 | 44.8 | 44.8 |
|  | S100A4 | 4.0 | 9.4 | 20.1 | 46.5 | 46.5 |
|  | α-SMA | 16.8 | 15.1 | 25.1 | 29.8 | 32.4 |
| **SASP** | CCL2 | 12.0 | 1.3 | 7.4 | 5.4 | 8.7 |
|  | IL-1β | 0.0 | 3.7 | 5.4 | 15.4 | 18.7 |
|  | IL-6 | 16.0 | 9.7 | 7.4 | 7.0 | 9.4 |
|  | IL-8 | 0.0 | 0.7 | 6.0 | 0.7 | 0.0 |
|  | TGF-β | 33.6 | 12.0 | 13.7 | 6.7 | 3.7 |
|  | Connexin 43 | 0.0 | 1.0 | 0.0 | 0.0 | 0.3 |
|  | PAI-1 | 0.0 | 0.0 | 3.3 | 1.7 | 1.0 |

**Zone definitions:** Zones were generated using a distance map approach based on the selected fibrosis niche, with increasing zone numbers representing increasing spatial distance from the reference niche**. Abbreviations:** CD, cluster of differentiation; EMT, epithelial-to-mesenchymal transition; SASP, senescence-associated secretory phenotype; HLA-DR, human leukocyte antigen-DR; 8-OHdG, 8-hydroxy-2′-deoxyguanosine; NOX4, NADPH oxidase 4; SOD2, superoxide dismutase 2; 53BP1, p53-binding protein 1; p16, cyclin-dependent kinase inhibitor 2A; p21, cyclin-dependent kinase 1A; p53, tumor protein p53; TIMP1, tissue inhibitor of metalloproteinases 1; ERRα, estrogen-related receptor alpha; SHH, sonic hedgehog; DDR1, discoidin domain receptor 1; α-SMA, alpha-smooth muscle actin; CCL2, C-C motif chemokine ligand 2; IL, interleukin; TGF-β, transforming growth factor beta; PAI-1, plasminogen activator inhibitor-1.
